# Sexually transmitted infections among HIV-infected women in Thailand

**DOI:** 10.1186/1471-2458-13-373

**Published:** 2013-04-22

**Authors:** Suvanna Asavapiriyanont, Rangsima Lolekha, Anuvat Roongpisuthipong, Amornpan Wiratchai, Surasak Kaoiean, Orapin Suksripanich, Amphan Chalermchockcharoenkit, Jaruensook Ausavapipit, Somporn Srifeungfung, Sarika Pattanasin, Kenneth A Katz

**Affiliations:** 1 Rajavithi Hospital, Bangkok, Thailand; 2 Thailand Ministry of Public Health - US Centers for Disease Control and Prevention Collaboration, Nonthaburi, Thailand; 3 Faculty of Medicine, Siriraj Hospital, Bangkok, Thailand; 4 Bamrasnaradura Infectious Diseases Institute, Nonthaburi, Thailand; 5 Global AIDS Program, US Centers for Disease Control and Prevention, Atlanta, GA, USA

**Keywords:** HIV-infected women, STI prevalence, Number needed to screen, Chlamydia, Gonorrhea, Thailand

## Abstract

**Background:**

Data on sexually transmitted infections (STI) prevalence among HIV-infected women in Thailand are limited. We studied, among HIV-infected women, prevalence of STI symptoms and signs; prevalence and correlates of having any STI; prevalence and correlates of *Chlamydia trachomatis* (CT) or *Neisseria gonorrhoeae* (GC) among women without CT and/or GC symptoms or signs; and number of women without CT and/or GC symptoms or signs needed to screen (NNS) to detect one woman with CT and/or GC overall, among pregnant women, and among women ≤25 years.

**Methods:**

During October 2004–September 2006, HIV-infected women at 3 obstetrics and gynecology clinics were asked about sexual behaviors and STI symptoms, physically examined, and screened for chlamydia, gonorrhea, trichomoniasis, and syphilis. Multivariate logistic regression was used to identify correlates of infections. NNS was calculated using standard methods.

**Results:**

Among 1,124 women, 526 (47.0%) had STI symptoms or signs, 469 (41.7%) had CT and/or GC symptoms or signs, and 133 (11.8%) had an STI. Correlates of having an STI included pregnancy and having STI signs. Among 469 women and 655 women with vs. without CT and/or GC symptoms or signs, respectively, 43 (9.2%) vs. 31 (4.7%), 2 (0.4%) vs. 9 (1.4%), and 45 (9.6%) vs. 38 (5.8%) had CT, GC, or “CT or GC”, respectively; correlates included receiving care at university hospitals and having sex with a casual partner within 3 months. NNS for women overall and women ≤25 years old were 18 (95% CI, 13-25) and 11 (95% CI, 6-23), respectively; and for pregnant and non-pregnant women, 8 (95% CI, 4-24) and 19 (95% CI, 14-27), respectively.

**Conclusions:**

STI prevalence among HIV-infected women, including CT and GC among those without symptoms or signs, was substantial. Screening for CT and GC, particularly for pregnant women, should be considered.

## Background

Sexually transmitted infections (STIs) can cause morbidity and increase risk of HIV acquisition and transmission [1]. For those reasons, the US Centers for Diseases Control and Prevention (CDC) recommends screening (defined as performing tests on persons without symptoms or signs of disease) of persons living with HIV/AIDS (PLHA) for STIs, including syphilis, gonorrhea, chlamydia, and (for women) trichomoniasis. Screening for those infections should occur at the first visit and should be repeated periodically thereafter, depending on the patient’s reported behaviors, the presence of STIs in the patient or the patient’s partner(s), and the STI prevalence in the community. HIV-care providers should also take a thorough sexual history and perform a physical examination, including for STIs, at the initial visit. Patients with STI symptoms or signs should be tested and managed appropriately [2-4].

In resource-limited settings, the World Health Organization (WHO) recommends that health-care providers obtain a thorough STI-related history from all newly diagnosed PLHA; conduct a physical examination to check for STIs; and perform serologic screening for syphilis and (for women) screening for gonorrhea and chlamydia. WHO recommends syndromic management of patients with STI symptoms or signs [5].

In Thailand, national guidelines recommend that providers take a history of STI symptoms and risk behavior of PLHA receiving care at each visit; perform screening for syphilis, but not for chlamydia and gonorrhea, at the initial visit and annually for PLHA with sexual risk behaviors; perform a Gram stain and bacterial culture of cervical discharge for symptomatic HIV-infected women; and manage STI symptoms or signs syndromically [6]. Because screening for gonorrhea and chlamydia is not recommended in Thai national guidelines, PLHA without signs or symptoms would miss an opportunity to receive treatment that can prevent damaging sequelae of those infections — including pelvic inflammatory disease, ectopic pregnancy, chronic pelvic pain, and infertility [7,8] — and can reduce risk of STI and HIV transmission to sex partners [1].

Limited data on STI prevalence and correlates among HIV-infected women in Thailand are available. Such data are necessary to guide national STI screening recommendations. Therefore, in this study, we investigated prevalence of STI symptoms and signs; prevalence and correlates of chlamydia and gonorrhea symptoms and signs among HIV-infected women; prevalence and correlates of having any STI among those women; prevalence and correlates of chlamydia or gonorrhea among HIV-infected women without chlamydia and gonorrhea symptoms or signs; and number of HIV-infected women (overall, and stratified by pregnancy status and age) without chlamydia and gonorrhea symptoms or signs needed to screen (NNS) to detect one woman infected with chlamydia and/or gonorrhea.

## Methods

During October 2004–September 2006, we conducted a cross-sectional study, enrolling consecutive HIV-infected women presenting for care at 3 obstetrics and gynecology (OB/GYN) clinics, including the university-affiliated Siriraj and Rajavithi Hospitals in Bangkok and the Bamrasnaradura Institute, a government-run infectious diseases hospital in Nonthaburi, the province north of Bangkok. Reasons for visiting clinics included antenatal care (ANC), CD4+ T-lymphocyte (CD4) monitoring and follow-up of women not eligible for antiretroviral treatment (ART), STI symptoms, partners of STI patients, referral from HIV clinic for Pap smear screening and reproductive health check-up, and HIV testing for partners of PLHA, people with sexual risk behaviors, or people with HIV-related symptoms. Counselors or nurses asked women about sexual behaviors, including history of vaginal or anal sex during last 3 months; condom use during last vaginal or anal sex; sex with casual partners, steady partners, or sex workers during the last 3 months; sex work during the last 3 months; and STI symptoms at time of consultation, including vaginal or urethral discharge, dysuria, lower abdominal pain, and genital pain or lesions. Symptoms of chlamydia and gonorrhea included all STI symptoms except genital pain or lesions. Data were recorded in a standardized format in patient records.

All consenting women underwent a genital and pelvic examination regardless of whether they reported STI symptoms. Signs of STIs were defined as genital wart, genital ulcer, inflamed cervix with or without pus or blood, yellow or green or white vaginal discharge, or adnexal or cervical motion tenderness during bimanual examination. Signs of chlamydia and gonorrhea included all STI signs except genital wart and ulcer.

STI treatment was done using a syndromic approach, according to Thai national guidelines, and was provided, along with condoms, free of charge.

### Laboratory evaluation

Endocervical specimens were obtained from all women for the detection of *Chlamydia trachomatis* and *Neisseria gonorrhoeae* using nucleic acid hybridization test (GenProbe Inc., San Diego, California, USA, at Siriraj and Rajavithi Hospitals) and nucleic acid amplification tests (NAATs) (Cobas Amplicor, Roche Diagnostic Systems, Basel, Switzerland, at Bamrasnaradura Institute). Serologic tests for syphilis were done using Rapid Plasma Reagin (RPR) card tests (New Market Laboratory, Kentford, UK) and, for specimens reactive on RPR testing, *Treponema pallidum* haemagglutination assays (TPHA; Fuji-rebio, Inc., Tokyo, Japan). Vaginal swab specimens were collected for saline wet preparations for detection of *Trichomonas vaginalis* by light microscopy.

Women were considered to have an STI if they had clinician-diagnosed genital ulcer disease (GUD); positive results for gonorrhea, chlamydia, or trichomoniasis; or syphilis (defined as RPR titer positive and a reactive TPHA test).

HIV infection status was confirmed by Murex HIV1-2 ELISA (Murex Biotech, Ltd., Dartford, UK) if not previously documented. CD4-cell count testing was performed using a FACScan flow cytometer (Becton Dickinson Immunocytometry Systems, San Jose, California, USA). Pregnancy status was assessed by a urine pregnancy test. Data on most recent CD4 count and viral load were obtained from medical records.

### Data analysis

Data were analyzed using STATA 11.0 (StataCorp., College Station, Texas, USA). Chi-squared tests and Wilcoxon rank sum tests were used for categorical and continuous data, respectively. Fisher’s exact test was used for categorical data when the expected frequency of any cell in 2x2 table was <5. For prevalence data, 95% confidence intervals (95% CI) were calculated using the binomial distribution. Factors associated with STIs and factors associated with chlamydia or gonorrhea among HIV-infected women without chlamydia and gonorrhea symptoms or signs were analyzed using bivariate and multivariate logistic regression to estimate odds ratios (OR) with 95% CI. All correlates from bivariate analysis with p <0.2 were included in a multivariate model, with a backward stepwise procedure used to include only variables with p <0.05 in the final model.

NNS was defined as the number of HIV-infected women without chlamydia and gonorrhea symptoms or signs who needed to be screened in order to identify one woman with chlamydia, gonorrhea, or chlamydia and/or gonorrhea [9]. We included the combined outcome of chlamydia or gonorrhea because most NAATs test simultaneously for both infections. We calculated NNS as the reciprocal of the prevalence (and 95% CIs, calculated using exact binomial methods) of chlamydia, gonorrhea, or chlamydia and/or gonorrhea among HIV-infected women without symptoms or signs. We calculated NNS for women overall, and for non-pregnant and pregnant women specifically, since chlamydia or gonorrhea in the latter group can be associated with additional adverse outcomes including preterm delivery, puerperal sepsis, and neonatal infections [7,10,11]. We also calculated NNS for women aged >25 years and ≤25 years, because women aged ≤25 years are at higher risk for chlamydia and gonorrhea and CDC recommends routine screening for chlamydia for all sexually active females aged ≤25 years [12].

This project was classified by the Thai Ministry of Public Health and CDC as a program activity, not requiring Institutional Review Board approval.

## Results

### Population

We enrolled 1,124 HIV-infected women, including 458 (40.7%), 300 (26.7%), and 366 (32.6%) from Siriraj Hospital, Rajavithi Hospital, and Bamrasnaradura Institute, respectively. Of those, 124 (11.0%) were pregnant, of whom 51 (41.1%), 71 (57.3%), and 2 (1.6%) were from Siriraj Hospital, Rajavithi Hospital, and Bamrasnaradura Institute, respectively. Patient demographics are summarized in Table 1. Median age was 32 years (interquartile range [IQR], 28-37 years) and the median time since learning HIV status was 3.2 years (IQR, 0.8-6.6 years). More than 60% of women at Siriraj (280/458 [61.1%]) and Rajavithi Hospitals (203/300 [67.7%]) presented for ANC, postpartum follow-up, or STI symptoms, whereas two-third (252/366 [68.9%]) of women at Bamrasnaradura Institute were referred from HIV clinics for annual Pap smear screening. Overall 521/1,124 (46.3%) were on antiretroviral treatment (ART), including 150/458 (32.8%), 88/300 (29.3%), and 283/366 (77.3%) at Siriraj Hospital, Rajavithi Hospital, and Bamrasnaradura Institute, respectively. The median CD4 cell count overall was 292 cells/mm^3^ (IQR, 140-453 cells/mm^3^). Among 521 women on ART, 247 women with viral load test results available, median viral load was <50 copies/mL (IQR, <50- < 50 copies/mL) (Table 1).

**Table 1:** Characteristics of HIV-infected women at three clinics in Thailand, according to presence or absence of chlamydia and/or gonorrhea symptoms or signs

**Characteristics**	**Number (%) of women**	Number with chlamydia or gonorrhea symptoms/signs*	Number without chlamydia or gonorrhea symptoms/signs*	**P-value**
	**(N = 1,124)**	**(n = 469, 41.7%) (95 % CI: 38.8-44.7)**	**(n = 655, 58.3%) (95% CI: 55.3-61.2)**	
**Demographic characteristics**				
Age, years (median (IQR))	32 (28-37)	31 (26-35)	33 (29-38)	<0.01
≤25	178 (15.8)	97 (20.7)	81 (12.4)	
>25	944 (84.0)	372 (79.3)	572 (87.3)	
Missing	2 (0.2)	0 (0.0)	2 (0.3)	
Hospital				
Siriraj Hospital	458 (40.7)	153 (32.6)	305 (46.6)	<0.01
Rajavithi Hospital	300 (26.7)	228 (48.6)	72 (11.0)	<0.01
Bamrasnaradura Institute	366 (32.6)	88 (18.8)	278 (42.4)	<0.01
Highest level of education, years (median (IQR))	9 (6-12)	7 (6-12)	9 (6-12)	0.30
≤ Grade 6	530 (47.1)	229 (48.8)	301 (46.0)	
> Grade 6	593 (52.8)	240 (51.2)	353 (53.9)	
Missing	1 (0.1)	0 (0.0)	1 (0.1)	
Years since HIV diagnosis (median (IQR))	3.2 (0.8-6.6 years)	2.7 (0.5-5.8 years)	3.4 (1.1-7.4 years)	0.02
Cause of HIV infection				
Heterosexual	1025 (91.2)	427 (91.0)	598 (91.3)	0.88
Other	99 (8.8)	42 (9.0)	57 (8.7)	
Pregnancy				
Yes	124 (11.0)	84 (17.9)	40 (6.1)	<0.01
**Clinical characteristics**				
Most recent CD4 count (cells/mm^3^)				
(median (IQR))	292 (140-453)	265 (110-438)	340 (160-460)	0.02
≤ 200	369 (32.8)	176 (37.5)	193 (29.5)	<0.01
> 200	744 (66.2)	285 (61.8)	459 (70.0)	
Unknown or missing	11 (1.0)	8 (1.7)	3 (0.5)	
Most recent HIV viral load: HIV-1 RNA (copies/mL) median (IQR)	<50 (<50- < 50)	<50 (<50- < 50)	<50 (<50- < 50)	
≤ 50	178 (15.8)	37 (7.9)	141 (21.5)	<0.01
> 50	69 (6.2)	33 (7.0)	36 (5.5)	
Missing	877 (78.0)	399 (85.1)	478 (73.0)	
Currently on antiretroviral treatment				
No	522 (46.4)	252 (53.7)	270 (41.2)	<0.01
Yes	521 (46.4)	171 (36.5)	350 (53.5)	
Unknown or missing	81 (7.2)	46 (9.8)	35 (5.3)	
**Behaviors in last 3 months**				
Sex in the last 3 months^1^	722 (64.2)	316 (67.4)	406 (62.0)	0.06
• Had steady partner^2^				
Yes	622 (86.2)	277 (87.6)	345 (85.0)	0.30
Condom at last sex	66.7%	59.3%	73.0%	
No	66 (9.1)	28 (8.9)	38 (9.3)	
Missing	34 (4.7)	11 (3.5)	23 (5.7)	
• Had casual partner ^3^				
Yes	29 (4.0)	12 (3.8)	17 (4.2)	0.79
Condom at last sex	56.0%	33.3%	76.9%	
No	639 (88.5)	291 (92.1)	348 (85.7)	
Missing	54 (7.5)	13 (4.1)	41 (10.1)	

^1^ Vaginal and/or anal sex.

^2^ Steady partner was defined as a person the woman has known for more than 2 months, with whom she has regular sex and to whom she feels psychologically connected.

^3^ Casual partner was defined as a person the woman has sex with and who do not meet the definition of CSW or steady partner.

*Chlamydia and gonorrhea symptoms or signs were defined as STI symptoms or signs except genital wart, genital ulcer, genital lesions.

### Prevalence of STI symptoms and signs, prevalence and correlates of chlamydia and gonorrhea symptoms and signs among HIV-infected women overall

Overall prevalence of STI symptoms or signs was 526/1,124 (47.0%, 95% CI: 43.8-49.8%) and prevalence of chlamydia and gonorrhea symptoms or signs was 469/1,124 (41.7%, 95% CI: 38.8-44.7%) (Table 2). Compared with women without chlamydia and gonorrhea symptoms or signs, women with symptoms or signs, respectively, were significantly more likely to be ≤25 years old (20.7% vs. 12.4%; P < 0.01), have received care at Rajavithi Hospital (48.6% vs. 11.0%; P < 0.01), had a more recent HIV diagnosis (2.7 years vs. 3.4 years; P = 0.02), be pregnant (17.9% vs. 6.1%; P < 0.01), had CD4 count <200 cells/mm^3^ (37.5% vs. 29.5%; P < 0.01), and not be receiving ART (53.7% vs. 41.2%; P < 0.01) (Table 1).

**Table 2:** Prevalence of STIs among HIV-infected women with and without STI symptoms or signs

				
**Laboratory-diagnosed sexually transmitted infections**	**Number (%, 95% CI) of women (N = 1,124)**	**Number (%; 95% CI) with**	**Number (%; 95% CI) without**	**P-value**
		chlamydia or gonorrhea symptoms/signs*	chlamydia or gonorrhea symptoms/signs*	
		**(n = 469) (41.7; 38.8-44.7)**	**(n = 655) (58.3; 55.3-61.2)**	
Chlamydia	74 (6.6; 5.2-8.2)	43 (9.2; 6.7-12.1)	31 (4.7; 3.2-6.6)	<0.01
Gonorrhea	11 (1.0; 0.5-1.7)	2 (0.4; 0.0-1.5)	9 (1.4; 0.6-2.6)	<0.01
Syphilis	8 (0.7; 0.3-1.4)	3 (0.6; 0.1-1.9)	5 (0.8; 0.2-1.8)	0.83
Trichomoniasis	17 (1.5; 0.9-2.4)	11 (2.3; 1.2-4.1)	6 (0.9; 0.3-2.0)	0.05
Any laboratory-diagnosed STI ^1^	106 (9.4; 7.8-11.3)	58 (12.4; 9.5-15.7)	48 (7.3; 5.4-9.6 )	<0.01
**Sexually transmitted infections**	**Number (%; 95% CI) of women (N = 1,124)**	**Number (%; 95% CI) with STI**	**Number (%; 95% CI) without STI**	**P-value**
		**symptoms/signs**^**#**^	**symptoms/signs**^**#**^	
		**(n = 526) (47.0; 43.8-49.8)**	**(n = 598) (53.0; 50.2-56.1)**	
Genital ulcer disease	28 (2.5; 1.7-3.6)	28 (5.3; 3.6-7.6)	0 (0.0; 0.0-0.6)	<0.001
Chlamydia	74 (6.6; 5.2-8.2)	47 (8.9; 6.6-11.7)	27 (4.5; 3.0-6.5)	<0.01
Gonorrhea	11 (1.0; 0.5-1.7)	3 (0.6; 0.1-1.7)	8 (1.3; 0.6-2.6)	0.19
Syphilis	8 (0.7; 0.3-1.4)	3 (0.6; 0.1-1.7)	5 (0.8; 0.3-1.9)	0.60
Trichomoniasis	17 (1.5; 0.9-2.4)	12 (2.3;1.2-3.9)	5 (0.8; 0.3-1.9)	0.05
Any STI^2^	133 (11.8; 10.0-13.9)	90 (17.1; 14.0-24.6)	43 (7.2;5.2-9.6 )	<0.001

^1^ Gonorrhea by NAAT/Chlamydia by NAAT/Trichomoniasis by wet mount/laboratory confirmed syphilis.

^2^ Gonorrhea by NAAT/Chlamydia by NAAT/Trichomoniasis by wet mount /laboratory confirmed syphilis/genital ulcer or GUD.

^#^STI symptoms or signs were defined as symptomatic vaginal discharge, urethral discharge, or lower abdominal pain or cervix examination (inflammation/pus/blood or other) or vaginal discharge on examination (white/yellow/green/other) or cervical motion tenderness during bimanual examination or genital wart, genital ulcer, genital lesions.

*Chlamydia and gonorrhea symptoms or signs were defined as STI symptoms or signs except genital wart, genital ulcer, genital lesions.

### Prevalence of STIs among HIV-infected women overall and pregnant women and correlates of women overall

Overall among 1,124 women, 133 (11.8%, 95% CI: 10.0-13.9%) had an STI and 106 (9.4%, 95% CI: 7.8-11.3%) had any laboratory-diagnosed STI. STIs included 74 (6.6%, 95% CI: 5.2-8.2%) with chlamydia, 28 (2.5%, 95% CI: 1.7-3.6%) with GUD, 17 (1.5%, 95% CI: 0.9-2.4%) with trichomoniasis, 11 (1.0%, 95% CI: 0.5-1.7%) with gonorrhea, and 8 (0.7%, 95% CI: 0.3-1.4%) with syphilis. Chlamydia prevalence was significantly lower and gonorrhea prevalence was significantly higher, among women without chlamydia and/or gonorrhea symptoms or signs compared with women with symptoms or signs (Table 2).

Among 124 pregnant women, 28 (22.6%, 95% CI: 15.6-30.9%) had an STI, including 22 (17.7%, 95% CI: 11.5-25.6%) with chlamydia, 4 (3.2%, 95% CI: 0.9-8.0%) with trichomoniasis, 2 (1.6%, 95% CI: 0.2-5.7%) with syphilis, 1 (0.8%, 95% CI: 0.0-4.4%) with GUD, and none with gonorrhea (0%, 95% CI: 0-2.9%).

In the final multivariate model, STI prevalence was significantly higher among pregnant women (adjusted odd ratio (aOR) 2.4, 95% CI: 1.4-3.9) and women who had STI signs (aOR 3.3, 95% CI: 2.2-4.9) compared with non-pregnant women and women without STI signs, respectively (Table 3).

**Table 3:** Factors associated with sexually transmitted infections among HIV-infected women at three clinics, Thailand (N = 1,124)

**Predictors**	**STI **^**1 **^**(No., %)**		**OR (95% CI)**	**P value**	**Adjusted OR**^**d **^**(95% CI)**	**P value**
	**Yes (n = 133)**	**No (n = 991)**				
**Age**						
≤ 25 yrs	30 (16.9)	148 (83.1)	1.7 (1.0, 2.6)	0.03	-	
**>** 25 yrs	103 (10.9)	841 (89.1)	1		-	
**Years of education**						
≤ Grade 6	62 (11.7)	468 (88.3)	1.0 (0.77, 1.4)	0.96		
> Grade 6	71 (12.0)	522 (88.0)	1			
**Hospital**						
Siriraj	59 (12.9)	399 (87.1)	2.1 (1.2, 3.6)	<0.01	-	
Rajavithi	50 (16.7)	250 (83.3)	2.8 (1.7, 4.9)	<0.01	-	
Bamrasnaradura	24 (6.6)	342 (93.4)	1		-	
**Pregnancy**						
Yes	28 (22.6)	96 (77.4)	2.5 (1.5, 4.1)	<0.01	2.4 (1.4, 3.9)	0.001
No	105 (10.5)	895 (89.5)	1		1	
**Sex work**						
Previous/ current	9 (16.7)	45 (83.3)	1.4 (0.6, 3.2)	0.44		
Never	115 (12.2)	831 (87.8)	1			
**Casual partner last 3 months**						
Yes	6 (17.6)	28 (82.4)	1.5 (0.5, 3.8) ^c^	0.43 ^f^		
No	119 (12.5)	833 (87.5)	1			
**Steady partner last 3 months**						
Yes	78 (11.9)	580 (88.1)	0.8 (0.6, 1.3)	0.43		
No	48 (13.8)	300 (86.2)	1			
**Condom use last 3 months**						
No	31 (13.4)	201 (86.6)	1.0 (0.6, 1.6)	0.92	-	
Yes	48 (9.8)	442 (90.2)	0.7 (0.4, 1.1)	0.11	-	
No sex last 3 months	54 (13.4)	348 (86.6)	1		-	
**Time after infection**						
**<** 6 months	31 (15.4)	170 (84.6)	1.4 (0.9, 2.3)	0.12	-	
**≥** 6 months	98 (11.2)	780 (88.8)	1		-	
**Latest CD4 count**						
0-200 cells/mm^3^	51 (13.8)	318 (86.2)	1.3 (0.9, 1.9)	0.21		
> 200 cells/mm^3^	82 (11.0)	662 (89.0)	1			
**Abnormal symptom **^2^						
≥ 1 symptom(s)	61 (17.3)	292 (82.7)	2.0 (1.4, 3.0)	<0.01	-	
None	72 (9.3)	699 (90.7)	1		-	
**STI signs **^3^						
≥ 1 sign(s)	73 (21.5)	266 (78.5)	3.3 (2.3, 4.9)	<0.001	3.3 (2.2, 4.9)	<0.001
None	60 (7.6)	725(92.4)	1		1	
**Latest viral load**						
<50 copies/ml	9 (5.1)	169 (94.9)	0.9 (0.2, 4.0) ^c^	0.76 ^f^		
≥50 copies/ml	4 (5.8)	65 (94.2)	1			
**Current ARV**						
No	72 (13.8)	450 (86.2)	1.5 (1.0, 2.2)	0.04	-	
Yes	50 (9.6)	471 (90.4)	1		-	

^f^ Fisher Exact test.

^c^ Exact Confidence Limits.

^d^ Adjusted for age, hospital, pregnancy, condom use last 3 months, time after infection, abnormal symptoms, STI signs, and current ARV treatment.

^1^ Gonorrhea by PCR/Chlamydia by PCR/Trichomoniasis by wet mount /GUD by doctor conclusion/laboratory confirmed syphilis (VDRL/TPHA).

^2^ Genital pain/ genital lesion/ vaginal discharge/urethral discharge/ lower abdominal pain.

^3^ Genital wart/genital ulcer/abnormal cervix/abnormal vaginal discharge/cervical motion tenderness during bimanual examination.

### Prevalence and correlates of chlamydia and gonorrhea among women with and without chlamydia and gonorrhea symptoms or signs

Among 469 women and 655 women with vs. without symptoms or signs, respectively, 58 (12.4%, 95% CI: 9.5-15.7) vs. 48 (7.3%, 95% CI: 5.4-9.6) had a laboratory-diagnosed STI, including 43 (9.2 %) vs. 31 (4.7%) with chlamydia and 2 (0.4%) vs. 9 (1.4%) with gonorrhea (Table 2). In the final multivariate model, chlamydia and/or gonorrhea prevalence was significantly higher among women receiving care at Siriraj Hospital (aOR 12.1, 95% CI: 2.8-52.0) or Rajavithi Hospital (aOR 20.3, 95% CI: 4.2-98.4), compared with women receiving care at Bamrasnaradura Institute; and among women having ≥1 casual partner during last 3 months (aOR 6.3, 95% CI: 1.6-24.7), compared with women having no casual partners during the last 3 months (Table 4).

**Table 4:** Factors associated with Chlamydia and/or Gonorrhea among HIV-infected women without chlamydia and gonorrhea symptoms and/or signs (N = 655)

**Characteristics**	**Chlamydia and/or Gonorrhea (No., %)**		**OR (95% CI)**	**P value**	**Adjusted OR**^**d **^**(95% CI)**	**P value**
	**Yes (n = 38)**	**No (n = 617)**				
**Age**						
≤25 yrs	8 (9.9)	73 (90.1)	2.0 (0.8, 4.7) ^c^	0.12 ^f^		
**>**25 yrs	30 (5.2)	542 (94.8)	1			
**Years of education**						
≤ Primary school	19 (6.3)	282 (93.7)	1.2 (0.6, 2.4)	0.73		
> Primary school	19 (5.4)	334 (94.6)	1			
**Clinic**						
Siriraj Hospital	26 (8.5)	279 (91.5)	12.9 (3.2, 112.5)	<0.01	12.1 (2.8, 52.0)	<0.01
Rajavithi Hospital	10 (13.9)	62 (86.1)	22.3 (4.5, 211.3)	<0.01	20.3 (4.2, 98.4)	<0.01
Bamrasnaradura Institute	2 (0.7)	276 (99.3)	1		1	
**Pregnancy**						
Yes	5 (12.5)	35 (87.5)	2.5 (0.7, 7.1) ^c^	0.07 ^f^	-	
No	33 (5.4)	582 (94.6)	1			
**Sex work**						
Previous/current^1^	4 (13.3)	26 (86.7)	2.4 (0.6, 7.6) ^c^	0.12 ^f^	-	
Never	32 (6.0)	503 (94.0)	1			
**Casual partner last 3 months**^2^						
Yes	3 (13.6)	19 (86.4)	2.4 (0.4, 8.7) ^c^	0.17 ^f^	6.3 (1.6, 24.7)	0.01
No	33 (6.2)	498 (93.8)	1			
**Steady partner last 3 months**^3^						
Yes	21 (5.8)	339 (94.2)	0.7 (0.4, 1.6)	0.52		
No	16 (7.6)	195 (92.4)	1			
**Condom use last 3 months**^4^						
No	10 (8.8)	103 (91.1)	1.6 (0.6, 4.1)	0.36		
Yes	14 (4.8)	279 (95.2)	0.8 (0.4, 1.9)	0.80		
No sex last 3 months	14 (5.6)	235 (94.4)	1			
**Time after infection**						
**<** 6 months	5 (5.3)	90 (94.7)	0.9 (0.3, 2.3) ^**c**^	0.97		
**≥** 6 months	32 (6.0)	503 (94.0)	1			
**Latest CD4 count**						
0-200 cells/mm^3^	10 (5.2)	183 (94.8)	0.8 (0.4, 1.8)	0.78		
> 200 cells/mm^3^	28 (6.1)	431 (93.9)	1			
**Latest viral load**						
≤50 copies/mL	9 (5.1)	169 (94.9)	0.9 (0.2, 4.0) ^**c**^	0.76 ^f^		
> 50 copies/mL	4 (5.8)	65 (94.2)	1			
**Current ARV**						
No	21 (7.8)	249 (92.2)	2.0 (1.0, 4.3)	0.07	-	
Yes	14 (4.0)	336 (96.0)	1			

^f^ Fisher Exact test.

^c^ Exact Confidence Limits.

^d^ Adjusted for clinic, pregnancy, sex work history, casual partner last 3 months, and current ARV treatment.

^1^ Current refer to sex work during last 3 months, Previous = Ever had sex work in the past but not practicing sex work during last 3 months.

^2^ Casual partner refer to a person that has sex with but do not fit in the definition of CSW nor steady partner.

^3^ Steady partner refer to a person that has been known for more than 2 months, having regular sex and feel psychological connected.

^4^ Vaginal and/or anal sex.

Denominator may vary due to missing data.

### Number needed to screen

**Figure 1:**
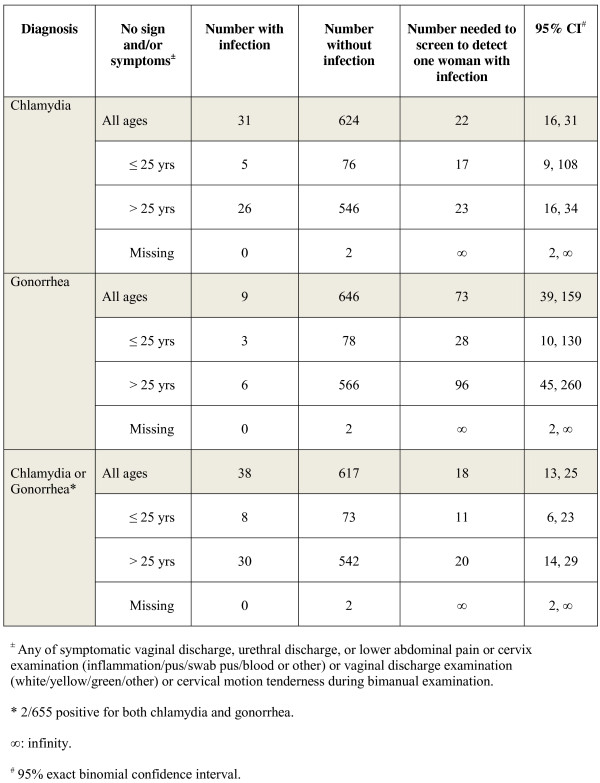
Number of HIV-infected women without chlamydia and gonorrhea symptoms or signs needed to screen to detect one woman with chlamydia, gonorrhea, or chlamydia or gonorrhea at three clinics in Thailand (N = 655).

Overall the NNS to detect one woman infected with chlamydia, gonorrhea, or chlamydia and/or gonorrhea were 22 (95% CI, 16-31), 73 (95% CI, 39-159), and 18 (95% CI, 13-25), respectively. NNS for women ≤25 years vs. >25 years for chlamydia, gonorrhea, or chlamydia and/or gonorrhea were 17 (95% CI, 9-108) vs. 23 (95% CI, 16-34), 28 (95% CI, 10-130) vs. 96 (95% CI, 45-260), and 11 (95% CI, 6-23) vs. 20 (95% CI, 14-29), respectively (Figure 1).

**Figure 2:**
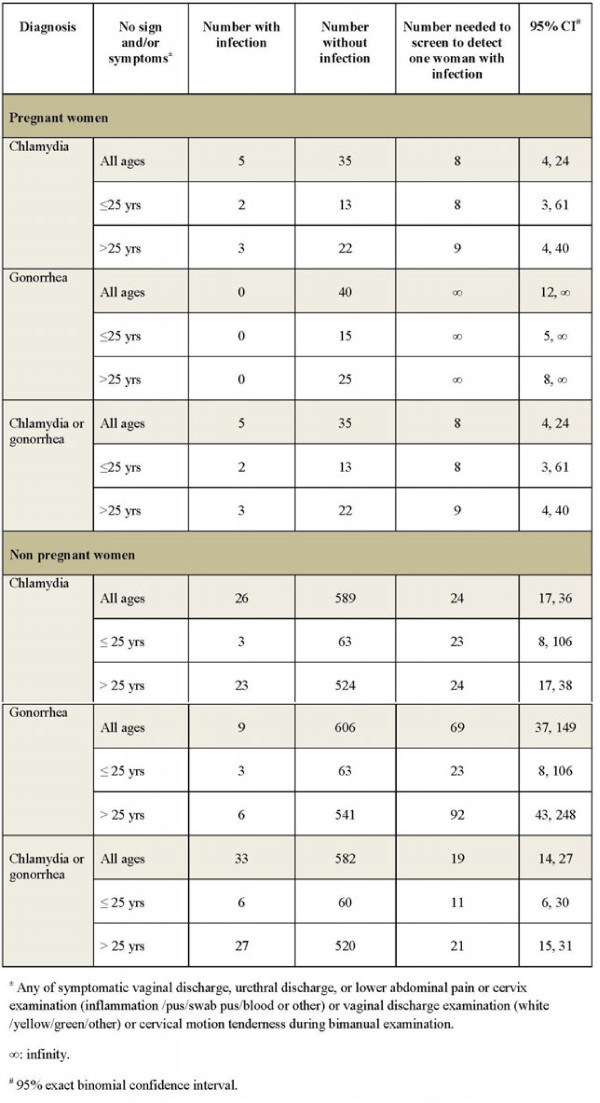
Number of HIV-infected pregnant and non pregnant women without chlamydia and gonorrhea symptoms or signs needed to screen to detect one woman with chlamydia, gonorrhea, or chlamydia or gonorrhea at three clinics in Thailand.

Among pregnant women, the NNS for chlamydia was 8 (95% CI, 4-24). Because no pregnant women had gonorrhea, NNS for gonorrhea could not be calculated (infinity) and the NNS for chlamydia or gonorrhea was equal to that for chlamydia alone. Among non-pregnant women, the NNS for chlamydia and/or gonorrhea was 19 (95% CI, 14-27), 11 (95% CI: 6-30), and 21 (95% CI: 15-31) for overall women, aged ≤25, and >25 years, respectively (Figure 2).

## Discussion

Substantial STI rate and low rate of condom use among HIV-infected women from our study highlighted the need for assessment of sexual risk behavior and STI symptoms or signs, particularly pregnant women at each clinical visit in order to identify women with risk for STI and provide appropriate evaluation and treatment. STIs in HIV-infected women when untreated may facilitate STI and HIV transmission to unprotected sex partners, contributing to new HIV and STI infections. Therefore, prevention with positives services should be emphasized and incorporated in routine service [2].

Although some studies in Europe and the U.S. have shown a low prevalence (≤5%) of STI among HIV-infected women [13-15], our study showed that STI prevalence among HIV-infected women (11.8%, 95% CI: 10.0-13.9%) was in the same range of many reports of HIV-infected women in Thailand and elsewhere (7-20%) [16-19]. Overall STI prevalence in women with STI symptoms or signs was higher than the prevalence in women without symptoms or signs. Although Thailand’s national HIV treatment and care guidelines recommend that providers take a history of STI symptoms and risk behavior at each visit and perform a Gram stain and culture of cervical discharge for symptomatic HIV-infected women, this study and other reports on HIV-infected women [20,21] and pregnant women [22] showed that some STIs including chlamydia, gonorrhea, trichomonas, and syphilis, can have no symptoms or signs. Using symptomatic screening for STI may not be sensitive enough to detect an STI among HIV-infected women. In contrast, women with STI symptoms or signs might not have an STI, since many of those symptoms and signs are not STI-specific. Therefore, clinicians should be aware that a recommendation for syndromic management for STIs among these women might lead to overtreatment. Simple and affordable STI diagnostic tests should be developed for use in routine STI screening of these women.

The overall chlamydia and gonorrhea prevalence among HIV-infected women in this study (6.6% and 1.0%, respectively) was not substantially different from the previous report of chlamydia and gonorrhea prevalence in HIV-uninfected Thai youth aged 15-21 years old (5% and 0.4%, respectively) [23]. Chlamydia prevalence among HIV-infected women without symptoms or signs in our study was lower than HIV-infected women with symptoms or signs 4.7% (95% CI: 3.2-6.6%) vs. 9.2% (95% CI: 6.7-12.1%). In this study, gonorrhea prevalence was higher among HIV-infected women without symptoms or signs (1.4%, 95% CI: 0.6-2.6%) than those with symptoms or signs (0.4%, 95% CI: 0-1.5%), in line with a report on gonorrhea in HIV-uninfected women [20]. This finding echoes those above that some STIs can present without symptoms and signs.

Chlamydia and gonorrhea prevalence among HIV-infected women without symptoms or signs receiving care at Bamrasnaradura Institute was significantly lower than chlamydia and gonorrhea prevalence among women receiving care at Siriraj or Rajavithi Hospitals. This may be due to the fact that the women receiving care at the 3 hospitals were from different population and may have had different expected risk for STIs. Almost 70% of women receiving care at Bamrasnaradura Institute’s clinic were referred there from HIV clinics for annual Pap smear and STI screening, including for chlamydia and gonorrhea, as part of an HIV quality improvement project [24]. So some HIV-infected women at Bamrasnaradura Institute were likely treated in the previous year for STIs detected prior to presentation at this visit. By contrast, Siriraj and Rajavithi Hospitals did not participate in the HIV quality improvement project.

Women without signs or symptoms who had sex with casual partners in the last three months were more likely to have chlamydia or gonorrhea than those who did not. This may be due to only half of these women reporting using condoms at last sex with casual partners.

In this study, pregnancy was associated with increased overall risk of STI. Having an STI during pregnancy can threaten the pregnancy and unborn baby’s health; some STIs can cross the placenta and infect the fetus or pass though the birth canal to cause peripartum infection [10,11]. The overall chlamydia prevalence among HIV-infected pregnant women was high (18%, 95% CI: 11.5-25.6%) and in line with a report on HIV-infected pregnant women in Thailand in 1997 (16%) [25]. The chlamydia prevalence in HIV-infected pregnant women was also higher than the prevalence among HIV-uninfected pregnant women in previous report in Thailand (9%) [25]. The low prevalence of gonorrhea is consistent with prior reports. No gonorrhea cases (0%, 95% CI: 0-2.9%) were detected among pregnant women in this study compared to previous reports of gonorrhea prevalence in HIV-infected pregnant women (2.7%) and HIV-uninfected pregnant women in Thailand (1.4%) [25].

This study showed that screening for chlamydia and gonorrhea for HIV-infected women without STI symptoms or signs can detect additional cases. NNS can be one of the metrics used to evaluate the cost-effectiveness of a potential or existing screening program. In this study, NNS for HIV-infected women, non-pregnant women, and pregnant women without STI symptoms or signs of chlamydia or gonorrhea was 18, 19, and 8, respectively. Because of additional potential benefits to the fetus, HIV-infected pregnant women might be the subgroup that benefits most from screening. For non-pregnant women, NNS for women aged ≤25 and >25 were 11 and 21, respectively. HIV-infected non pregnant women aged ≤25 years who have unprotected sex may be the subgroup that benefits second-most from screening.

Our study is one of a few studies in Thailand that reported STI prevalence among HIV-infected women [17,25-27]. It is the first study to report NNS among HIV-infected women and HIV-infected pregnant women in Thailand without STI signs and symptoms. This study has at least four limitations. First, the data are from 2004-2006 and might not represent the current situation, in which ART has become more available and prevention with positives programs have been recommended as standards [28]. Second, the data are limited to only HIV-infected women seen at tertiary care facilities in Bangkok and Nonthaburi and might not be generalizable to other treatment settings or geographic areas in Thailand. Third, we did not collect data for the total number of women who were approached to participate in this project. Study nurses estimated that 10-20% of approached women declined to participate because of they were uncomfortable talking about STIs or believed they did not have an STI because they were asymptomatic and/or on ART. Finally, we did not collect data on other STIs (e.g. herpes simplex virus infection, hepatitis B virus infection), and bacterial culture from women with STI symptoms or signs for confirmation of laboratory-diagnosed STIs.

## Conclusions

Overall STI prevalence among HIV-infected women seen at three OB/GYN clinics in Thailand during 2004-2006 was 11.8%. Clinicians should be aware of high rates of STIs, particularly among pregnant women and women with STI symptoms or signs. Using STI symptomatic screening and syndromic management alone might result in missed opportunities to detect STIs and to overtreatment of STI-uninfected women. Based on findings from this study, chlamydia and gonorrhea screening for HIV-infected women, including pregnant women, can detect additional STIs. In order to evaluate the utility of screening for chlamydia and gonorrhea among HIV-infected women, more studies are needed regarding the cost-effectiveness of such screening, including projected screening costs and benefits.

## Competing interests

The authors declare that they have no competing interests.

## Authors’ contributions

SA and RL participated in study design, project implementation, statistical analysis, interpretation of data, and drafting and revision of the manuscript. AR, AW, SK, OK, AC, JA, SS participated in study design, project implementation, and data collection. SP performed data analysis. KK participated in study design, statistical analysis, interpretation of data, and revision of the manuscript. All authors reviewed and approved the final version of the manuscript.

## Pre-publication history

The pre-publication history for this paper can be accessed here:

http://www.biomedcentral.com/1471-2458/13/373/prepub
